# A Speculative History of DNA: What If Oswald Avery Had Died in 1934?

**DOI:** 10.1371/journal.pbio.2001197

**Published:** 2016-12-22

**Authors:** Matthew Cobb

**Affiliations:** School of Biological Sciences, University of Manchester, Manchester, United Kingdom

## Abstract

This speculative Essay explores the consequences of the imagined premature death of Oswald Avery, who in 1944 provided evidence that genes are made of DNA. Four imaginary alternate routes to the genetic function of DNA are outlined, each of which highlights different aspects of the actual process of discovery.

## Introduction

In 1934, Oswald Avery (1877–1955), a Rockefeller Institute microbiologist, underwent surgery for Graves disease. Avery recovered from his thyroidectomy and returned to the laboratory, where he began trying to identify the “transforming principle.” This substance, produced by *Pneumococcus* bacteria, enabled harmless, rough bacteria to be transformed into virulent smooth bacteria, and vice versa [1,2,3]. In February 1944, after nearly a decade of painstaking experimentation, Avery and his coworkers Maclyn McCarty and Colin McLeod published an article in the *Journal of Experimental Medicine* showing that the transforming principle was made of deoxyribonucleic acid [4]. They suggested that this identification of what looked like a gene might be applicable to other organisms. Supporting evidence from *Escherichia coli* was rapidly provided by André Boivin in France [5], and soon scores of researchers around the world embraced the implication: genes are made of DNA [6].

That is history as it happened. But in an alternate reality, something went wrong on the operating table, and Avery died. His laboratory was closed, neither McCarty nor McLeod carried out their analytical work, and the transforming principle was not identified as DNA. What would have happened to science? Without Avery, how, where, and when would we have discovered what genes are made of?

This kind of counterfactual musing, often typical of late-night bar discussions at conferences, can have a serious side: it has been harnessed to help genetics students understand the origins of their subject and appreciate the sources of concepts they think are self-evident [7]. Some historians consider a deep exploration of counterfactuals to be a useful tool in exploring how and why events unfolded [8], although others disagree [9]. The purpose of this essay, however, is not so earnest; it is primarily intended to provoke and entertain and to reveal the swirling confusion that surrounded past views of what we now consider to be obvious. The speculations that follow are not the only possible alternatives nor are they unchallengeable—the reader may easily come up with others or see problems with the alternative histories outlined here.

## The Importance of Avery

Avery was not the only person interested in pneumococcal transformation in the 1930s, but the two other people working in the field, Fred Griffith in London [3] and Fred Neufeld in Berlin [10], both died in the Second World War. Even if one of them had survived, the fact that they did not attempt to identify the material basis of transformation in the 1930s suggests that they did not have either the necessary laboratory facilities or the required intellectual ability or appetite.

Although DNA was known to be a major component of chromosomes, in the 1930s nobody imagined there was a link between genes and DNA. In 1936, the crystallographer Dorothy Wrinch voiced the predominant view when she suggested that genes were made of proteins, interwoven in a DNA structure [11]. In the same year, Wendell Stanley showed that the active component of the tobacco mosaic virus was a protein (in 1946, he won the Nobel Prize for this mistaken claim). Viruses were widely assumed to be similar to genes, so this “discovery” reinforced the supposition that genes were made of proteins. DNA, on the other hand, was thought to be “boring” because it was composed of four bases, probably in equal proportions. There seemed to be no way that DNA could have what was called “specificity,” i.e., the ability to exert a vast range of extremely precise effects, as genes were able to. Proteins, it was well known, could be massively variable in their structure, much as genes could function in a huge variety of ways.

This protein-centred view of genes helps explain why Avery’s 1944 paper was greeted with skepticism by some researchers and was not immediately embraced by the whole scientific community [6]. It took much longer than most people now realise for the genetic role of DNA to be completely accepted: as late as 1961, an article in *Nature* acknowledged the possibility that genes might be made of proteins [12]. In reality, the road to accepting the hereditary role of DNA was extremely rocky. In order to provoke the reader into thinking about how we know what we know about the genetic role of DNA, I imagine four broad alternative timelines, each without Avery and each dominated by the protein-based conception of genes that his work overthrew. It seems probable that all of these paths would have involved an even more complex and slow route to the truth than the history we know.

## Avery without Avery

Avery’s work involved an experimental proof of the role of DNA in heredity. A simple route to understanding the function of DNA in the absence of Avery’s research would be to imagine someone else carrying out the same kind of study. Although there were no competitors able to pursue this approach, Avery’s untimely death could have inspired either his younger brother, Roy, or his colleague and flatmate Alphonse Dochez to develop Avery’s work in his place. This is not entirely fanciful. Roy Avery was a Vanderbilt University microbiologist and was close to Oswald—in May 1943, Roy was the first person outside of his brother’s laboratory to learn that the transforming principle was made of DNA [2]. Dochez, on the other hand, had collaborated with Avery on serological methods for identifying different types of pneumococcus, and their domestic and professional conversations must have included discussions of transformation and its physical basis.

In this version of history, either Roy Avery or Dochez identified the transforming principle as DNA, on a similar timescale to Oswald Avery, inspiring Erwin Chargaff, André Boivin, Joshua Lederberg, and others to focus their attention on this molecule, pretty much as actually happened [13]. It would not even be unreasonable to imagine that, at the end of this path, Jim Watson, Francis Crick, Maurice Wilkins, and Rosalind Franklin would be waiting to play their decisive roles.

Another possibility would be that perhaps one of the many biochemists who was studying chromosome structure and function would have eventually shown that DNA was the hereditary material. Perhaps, in a delicious irony, it would have been Avery’s Rockefeller colleague Alfred Mirsky, who in reality vigorously opposed Avery’s interpretation and argued that the supposed effects of nucleic acids in the transforming principle were due to minute protein contamination [14].

However, for someone outside of Avery’s circle to have carried out such a study, they would have had to find an appropriate biological system. Pneumococcal transformation was striking but obscure, and it seems unlikely that anyone but a skilled microbiologist would either be aware of the phenomenon or have the necessary ability to investigate it. Determining the genetic role of DNA in organisms apart from *Pneumococcus* proved difficult for Avery’s supporters in the 1940s and 1950s: Boivin’s results from *E*. *coli* were not widely accepted, and no other species was successfully manipulated at this time. This suggests that it would be unlikely that a successful alternative system would have popped up in a different timeline.

The most obvious alternate path would have involved one of the researchers working on bacteriophage carrying out a routine experiment to confirm Stanley’s suggestion that proteins were the determinant part of a virus, by separating the roles of DNA and protein in viral reproduction. For many students—and perhaps for many readers—that is in fact what happened, in the shape of Al Hershey and Martha Chase’s “blender” experiment, which was published in 1952, eight years after Avery’s paper appeared.

However, despite what the textbooks claim, it has long been known that Hershey and Chase’s experiment was so full of contamination that it did not in fact show that DNA was the genetic material in viruses, nor did Hershey suggest that it did [15,16]. Indeed, a year later, after the discovery of the double helix, Hershey presented his data at Cold Spring Harbor and argued that DNA was probably not the sole determiner of heredity [17].

Furthermore, one of the reasons Hershey did the experiment was that he was interested in Avery’s results. He had closely followed the work of the Avery lab, and in 1950 and 1951, several of his colleagues in the “phage group” repeatedly wondered whether the phage protein component acted like a hypodermic syringe, injecting the DNA transforming principle into the cell [13]. Without Avery’s finding and the acceptance by an important part of the scientific community that genes were made of DNA, it might have taken even longer for a blender-type experiment to be performed.

Nevertheless, given the widespread postwar interest in viruses and viral reproduction, it seems inevitable that something like the Hershey and Chase experiment would eventually have been carried out. The researchers would have had to obtain cleaner, more impressive results than Hershey and Chase were able to produce because in the absence of Avery’s findings, data of the quality provided by Hershey and Chase would not have been convincing. Then, in order to show that the effect was not limited to viruses, they would have had to extend the role of DNA to actual genes, in an organism. In the absence of an amenable system, it might have taken years before the link between the role of DNA in viral reproduction and its function in organismal genetics was demonstrated.

## From Structure to Function

A radically different route to revealing the genetic role of DNA would have proceeded in the opposite direction to that which actually occurred. In reality, Avery discovered the function of DNA, which led researchers to try and discover the molecular structure that could explain that function. Following theoretical speculation in the late 1940s by Masson Gulland [18] and Erwin Chargaff [19] that the sequence of the bases in DNA might not be uniform, and their discovery of the double helix structure of DNA, in 1953 Watson and Crick put forward the hypothesis that “the precise sequence of the bases is the code which carries the genetical information” [20]. This brilliant suggestion implied that the sequence of bases could encode infinite variability—Rosalind Franklin independently realised the same thing in early 1953 [21].

An alternative history could have involved X-ray crystallographer William Astbury. In reality, Astbury’s PhD student, Florence Bell, made the first X-ray crystallography images of DNA in 1936, and Astbury was an enthusiastic follower of Avery’s work [22]. Astbury’s research on DNA eventually petered out in the 1950s for complex reasons, not least of which was a lack of funding [23,24]. In an alternate timeline, Astbury could have successfully pursued his study of the structure of DNA, right up to the double helix. His interest in DNA would have been based on its apparent structural significance in chromosomes; the molecule might also have appeared to be a relatively simple structure that could be studied with X-ray crystallography.

It might seem counterintuitive, but it is even just possible that, in a world dominated by the idea that genes were made of proteins, the discovery of the double helix, and of complementary base-pairing, would not necessarily have led to the realisation that the DNA molecule can encode genetic information. If it were thought that the molecule merely had a structural function in chromosomes, it could have been assumed that the order of the bases was repetitive. Without experimental evidence of a genetic function for DNA, the potential of variation in base sequence to encode information could have been overlooked for some time. One argument against this would be that once the amino acid sequence of the first proteins was described, attention would inevitably have turned to potential variability in nucleic acid sequences. However, in the absence of decisive evidence that DNA was the genetic material, such as that provided by Avery, the final stage of such a timeline could conceivably have relied upon the development of the ability to sequence nucleic acids, which in real history did not occur until the Nobel Prize-winning work of Robert Holley in the 1960s (tRNA) and of Fred Sanger and Wally Gilbert in the 1970s (DNA).

Although unlikely, this suggests that in a world dominated by a protein-centred view of genes, the structure of the double helix could have been known and even fêted—complementary base-pairing would have helped explain how chromosomes replicate—but the fine detail of any given DNA molecule, and the information that contained, could have remained unsuspected.

## Protein Synthesis

Perhaps the most experimentally complex route to the genetic function of DNA would have involved following in reverse the flow of information identified by Crick in his “central dogma” lecture of 1958: protein → RNA → DNA [25]. In 1941, George Beadle and Ed Tatum suggested that each gene might control a particular biochemical reaction by synthesising an enzyme [26]. In an alternate history, studying protein synthesis could eventually have led back to the physical basis of the gene. By the mid-1940s, Torbjörn Caspersson in Sweden and Jean Brachet in Belgium had shown that when cells were actively synthesising proteins, RNA levels increased [27,28]. Much of this RNA was probably ribosomal, but by the end of the 1940s, more transient RNA species (mRNA) had been discovered, although their significance was unclear [29].

It is quite possible that this work could have occurred in the context of a protein-centred view of the gene; indeed, in the earliest phases, it did. However, the discovery of the function of mRNA was complex, and it would inevitably have been even more so if mRNA were thought to be an intermediary between a protein-based gene and a protein gene product. Sooner or later, and probably sooner, this idea would have seemed intrinsically unlikely. Once it was realized that mRNA encoded genetic information by the sequence of its bases, then scientists would immediately have asked why DNA could not do the same. In a world without Avery, the race to understand the nature of the hereditary material might have begun with such an insight.

## Convergent Evidence

In real history, there was no single moment when scientists realised that genes were made of DNA. For some, Avery’s work provided the key evidence; for others, it was Hershey and Chase’s experiment or the discovery of the double helix. At the level of the scientific community as a whole, all this evidence contributed to the growing acceptance in the 1950s of the “working hypothesis” that genes in all organisms (not just bacteria and viruses) were made of DNA [30,31].

In 1950, Daniel Mazia highlighted two key experimental criteria that any substance thought to be the physical basis of the gene would have to meet: there had to be the same amount of the substance in every diploid cell of a given species and that amount had to double in mitosis and be halved during meiosis [32]. Even with the relatively crude analytical techniques available at the time, it was obvious that no protein or group of proteins met these criteria—this was partly what led Mazia to reluctantly embrace the genetic role of DNA.

In a scientific world convinced of the genetic role of proteins, this kind of rigorous examination of data and sifting of experimental facts would still have taken place and would eventually, in the mind of some iconoclast, have led to the heretical suggestion that DNA was the genetic material. That insight would then have required experimental proof and structural evidence from investigations of the nature of nucleic acids, as occurred in real history.

## Conclusion

The alternative timelines here each underline, in different ways, the way that history actually took place and the complex set of proofs and contexts that were required for scientists to accept that genes were made of DNA. They also show that, in the absence of a nucleic-acid based view of heredity, even an iconic discovery such as the double helix structure of DNA would not necessarily have the immediate implications that we now assume it has. In the absence of sequence data showing variability in the order of the bases or of experimental proof of the genetic role of DNA, the ability of the double helix to encode genetic information would not necessarily have been immediately obvious.

In all these speculative scenarios, science would have returned to something like its current trajectory. Because the genetic role of DNA is knowable and of intrinsic interest, had Avery not lived to carry out his work, someone else would inevitably have discovered that genes are made of DNA. The alternative histories explored here do not give rise to immense changes in the timeline or to a strange alternate universe. Nonetheless, as well as providing a distracting look at history, they also help us to understand how discovery took place and emphasize that many of the ideas we now consider to be self-evident were anything but so in the time of the pioneers.
